# The practice, nature, and impact of nurse-led type 2 diabetic foot prevention services and educational programs in Sub-Saharan Africa: a scoping review

**DOI:** 10.3389/fpubh.2024.1465750

**Published:** 2024-11-25

**Authors:** Rincy Sajith, Louise Ackers, Simona Ackers-Johnson, Daniel J. Parker, Melanie Stephens

**Affiliations:** ^1^School of Health and Society, University of Salford, Salford, United Kingdom; ^2^Wear Rivers Trust, Durham, United Kingdom

**Keywords:** type 2 diabetes, nurse-led, foot prevention, LMIC, Sub-Saharan Africa, chronic disease management, public health, diabetes self-management education (DSME)

## Abstract

**Objective:**

The objective of this study is to assess the scope of existing practice, nature, and impact of nurse-led type 2 diabetic foot prevention services and educational programmes in Sub-Saharan Africa (SSA).

**Introduction:**

Type 2 diabetes mellitus (T2DM) in SSA imposes a heavy burden on current healthcare services. Complications such as foot ulcers can have a significant impact on patient care and healthcare resources. It is imperative to identify patients at risk of developing diabetic foot complications and empower them with diabetes self-management education and support from specialised foot clinics is crucial. However, the availability of such programmes and services in SSA is limited.

**Inclusion criteria:**

Studies of nurse-led diabetic foot prevention services and/or educational programmes in low- or middle-income countries in SSA for adults with T2DM, written in English, between August 2013 and March 2024 were considered.

**Methods:**

Following the standard Preferred Reporting Items for Systematic Reviews and Meta-Analyses (PRISMA) guidelines for conducting and reporting scoping reviews, searches were conducted on four electronic databases (CINAHL, ProQuest, MEDLINE, and Scopus) and Google Scholar. The titles and abstracts were scrutinised. All eligible papers were retrieved and screened for full text.

**Results:**

The review included ten studies (across 14 papers), all of which focused on nurse-led diabetes self-management education (DSME) programmes in SSA. There are no specific educational programmes or services led by nurses that focus exclusively on diabetic foot prevention. The analysis highlighted the components of successful nurse-led DSMEs that led to positive glycaemic control and self-care behaviors, including the focus on behavior change and the DSME should be co-produced with service users. The theoretical aspects of the DSME include evidence-based, structured, interactive, culturally and linguistically appropriate group-based activities. The DSME should be delivered over a period of several weeks, and sessions should last between 1.5 and 2 h. Barriers to delivery and participation include the rainy season, stockouts, time and resources needed, and a DSME that meets diverse levels of literacy and education.

**Conclusion:**

There is a heightened need for nurse-led, co-produced, culturally congruent, frugal, and sustainable education interventions or programmes. There is also a need for diabetic foot screening and foot ulcer prevention services that can operate sustainably alongside these educational interventions through task-shifted, simple, and frugal initiatives.

## Introduction

As one of the four primary types of non-communicable diseases (NCD), diabetes has emerged as an epidemic and a global health care issue (1). People of all age groups and countries are affected. However, the prevalence of adults with type 2 diabetes mellitus (T2DM) in Sub-Saharan Africa (SSA) is projected to increase from 23.6 million in 2021 to 54.9 million people in 2045 (2). In low- or middle-income countries (LMICs) across SSA, contributing risk factors resulting from unplanned urbanisation, globalisation of unhealthy lifestyles, and an ageing population are leading to diabetes-related health complications and premature deaths (1). In SSA, diabetic foot ulcers (DFUs) represent the largest proportion of admissions, amputations, and mortality. In a systematic review and meta-analysis of 55 studies and 10 abstracts, Rigato et al. (3) reported that 15% of patients with foot ulcers underwent major amputation, and 14.2% died during hospitalisation. Despite this, Manne–Goehler et al. (4), through a pooled analysis of 12 cross-sectional surveys, demonstrated that diabetes diagnosis and care remain largely unmet in SSA. It is important to note that a large proportion of adults with diabetes remain undiagnosed and that healthcare services are only accessed once complications such as DFUs have developed. Therefore, a call for policies and programmes to increase awareness and access for adults with low educational attainment and income from health care policy makers in SSA surrounding T2DM is needed.

The socioeconomic impact of NCDs, such as T2DM, is closely linked to poverty in LMICs, affecting not only poverty reduction initiatives and healthcare resources but also household-associated healthcare costs too (1). A method of controlling NCDs such as T2DM and their health-related complications is to focus on reducing risk factors such as detection, screening, education, and treatment. Education and lifestyle modification programmes that promote self-care practices, coupled with effective screening programmes that include evaluation of the vascular and neuropathy status, may play a pivotal role in countering diabetic foot disease and reducing diabetes morbidity and mortality in Africa (3, 5).

In high-income countries, structured diabetes self-management education (DSME) programmes are associated with improved outcomes, such as HbA1c, self-efficacy, and diabetes knowledge (6). These programmes are frequently delivered by multidisciplinary teams and are offered alongside other diabetes healthcare services such as podiatric-led screening and treatment services. However, in LMICs, the current evidence base for the impact of DSME’s is limited to short-term HBA1c control and behavior change. This is due to differences in delivery of the DSMEs, such as only 57% being culturally and linguistically tailored, differences in length of contact time with participants, and training of providers for adults with low level literacy (7). A recent multicentre randomised trial conducted by Lamptey et al. (8) across two hospitals in Ghana revealed that a UK-adapted DSME was not associated with glycaemic control in adults living with T2D. The researchers noted that, despite delivering a culturally tailored programme for adults with low levels of literacy, deprivation restricted the options of acting on lifestyle changes for the participants. They recommended future studies in resource-constrained settings that observe the long-term durability of the DSME. The DSME was also delivered over a period of 6 h, which could also have been a factor in ensuring long-term behavior change.

In SSA, there is an increasing need to leverage available health care workers to provide care for non-communicable diseases (9). Literature from historical studies in HIV (10, 11) and more recently in diabetes (12) demonstrated that nurses who work in resource-constrained services can manage stable patients’ care when following standardised protocols and guidelines to relieve the healthcare gap in LMICs. Kavita et al. (13) further support this by examining the effectiveness of interventions led by nurses in LMICs in a systematic review and meta-analysis. The authors identified 16 studies focusing on diabetes and 4 addressing diabetes and hypertension. The LMICs encompassed nations such as India, Brazil, Iran, Turkey, and Thailand, with findings indicating a significant reduction in HbA1c and Fasting Blood Sugar. The review highlighted that, whereas the impact is positive, scaling up of nurse-led interventions for NCDs needs to be increased. This highlighted a gap in LMICs in comparison to HICs, which use nurse practitioners in independent roles. Kumah et al. (14) conducted a scoping review to examine if differences in DSME outcomes were affected by the model of care and the type of health care provider (general practitioner, a specialist, a nurse, or a combination of these health professionals). The authors reported that DSMEs that foster effective collaboration between patients’ care providers and self-management instructors reported better outcomes, but conclusions on models of care could not be reached. Furthermore, the authors also reported that, due to a complete lack of studies in SSA in particular, more research was needed.

The Ugandan Ministry of Health’s Human Resources for Health (HRH) Strategic Plan (2020–2030) highlights the significance of task-shifting towards efficient human resource management and service delivery. Citing World Health Organization (15, p.389), it defines Task Shifting as, *‘the rational distribution of tasks among health workforce teams, with specific tasks moved from highly qualified health workers to health workers with shorter training and fewer qualifications in order to make efficient use of the available resources’.* Task-shifting, especially in low-resource settings, is advocated by the World Health Organization (9, 15–17) as a mechanism to improve health system efficiency and reduce the impact of severe health worker shortages. Shifting the focus from ‘treatment’ to ‘prevention’ implies a greater role for those health workers located closer to citizens, predominantly in community settings. In Uganda, this implies a strong emphasis on nurses.

A quality improvement project in Tanzania by Hall et al. (18) examined whether diabetes foot risk screening and patient education regarding foot care could be implemented in a nurse-led diabetes clinic of a busy outpatient centre in a LMIC. The research team discovered that more than 25% of patient visits were shown to have complete foot screening exams and patient education documented at four months post interventions. They concluded that contextually appropriate preventive guidelines could be effectively implemented in nurse-led clinics. A needs analysis survey conducted by Kuguyo et al. (19) revealed inadequate interventions for diabetic foot care in Zimbabwe, due to a dearth of podiatrists, foot screening services, and education and training for health care providers. The authors concluded that further research was needed to assess the knowledge and presence of training programmes to improve foot care for adults living with diabetes across Zimbabwe and LMICs and was urgently needed. In response to the need to curb diabetic foot disease, Manickuma et al. (20) conducted a RCT in Ghana that aimed to determine the effectiveness of a foot-care education module on changes in knowledge and behavior in adults with T2DM. Although their study was not designed to examine the impact of nurse-led intervention, the researchers recruited two nurses to assist in the development and delivery of the module. Participants were randomly assigned to one of three groups, and all received baseline treatment. Group 1 received no further treatment. Group 2 received a footcare handout with instructions to follow. Group 3 received a teaching session, a foot-care handout with instructions and pictures on practices to follow, and five lower-limb exercises. A pre- and post-test questionnaire was used to assess knowledge and behavior change across the groups. Significant changes in foot care behaviors were observed in Groups 2 and 3, and knowledge transfer of the lower limb exercises was observed in Group 3. Although the study demonstrated the viability of a simple pamphlet and educational programme to foot care practices of adults with T2DM, the study was small, in a single centre, within a 6-week period and did not follow the participants up longer term in regard to retained knowledge, practice, and prevalence of foot complications/ulcers.

Previous systematic or scoping reviews of empirical studies conducted in LMICs of SSA have measured the impact of DSMEs using outcomes such as glycaemic control (7), individual participation in self-management practices (21), the type of educational intervention used (22), and the place delivered (e.g., in primary care settings) (23). However, evidence of differences in DSME outcomes when the programme is nurse-led in SSA in comparison to other LMICs has yet to be established (14). This includes an exploration of the typology of DSMEs and their impact on primary and secondary outcomes (short- and long-term). These gaps in the literature and the aim of the research team to develop a DSME for Uganda as part of a Burdett Nursing Trust Grant have led to the primary objective of this review which is to assess the extent and type of evidence on the impact of nurse-led type 2 diabetic foot prevention services and educational programmes in SSA on secondary outcomes which include clinical outcomes (HbA1c or glycated haemoglobin or A1c, blood pressure, weight, and so on), psychosocial and behavioral outcomes (healthy eating, taking medications, being active, etc.), patient-reported outcomes (Health-related quality of life, self-efficacy, diabetes distress, patient satisfaction) and patient generated health data (blood glucose trends, steps taken, sleep, etc.). A preliminary search of MEDLINE, the Cochrane Database of Systematic Reviews, and JBI Evidence Synthesis was conducted, and no current or ongoing systematic reviews or scoping reviews on the topic were identified. To address this gap in the literature, a scoping review was selected as appropriate with the purpose of identifying the types of available evidence and identifying and clarifying key characteristics and outcomes of nurse-led education programmes in SSA, as indicated by Munn et al. (24).

### Research question and objectives

The research question for this scoping review was: what is known about the scope of existing practice, nature, and impact of nurse-led diabetic foot prevention services and structured educational programmes on patients with Type 2 Diabetes Mellitus in Sub-Saharan Africa? Through answering this question, we intend to address the following objectives: To assess the level of evidence for nurse-led diabetic foot prevention programmes and education programmes for type-2 diabetes in Sub-Saharan Africa; to examine whether there are different types of nurse-led diabetic foot prevention services and structured educational programmes; to identify facilitators and/or barriers that have been reported relating to the success and/or failure of the services/programmes; and to assess the impact of the services/programmes on patients and staff.

## Methods

This scoping review was conducted in accordance with the JBI methodology (25). The PRISMA extension for Scoping Reviews (PRISMA-ScR) was used to report the review findings (26).

This review followed the Participant, Concept, Context (PCC) approach to develop eligibility criteria. Participant Inclusions: The participants analysed were adults with type-2 diabetes mellitus; Participant Exclusions: Studies that reported participants without diabetes or non-type 2 diabetes; and studies that included children. The concept being explored pertains to nurse-led diabetic foot prevention and education programmes. Leadership was assessed by studies that reported that registered nurses led the delivery of the DSME or foot prevention programmes. The concept exclusions included studies that were not conducted by nurses and those that examined alternative self-management prevention and education programmes. The context encompassed Sub-Saharan African nations that were classified as low- or middle-income countries. The context exclusions included non-Sub-Saharan African countries that were classified as upper middle-income or high-income countries. The sources deemed eligible for this review comprised peer-reviewed primary research studies encompassing both experimental and quasi-experimental study designs, including randomised controlled trials (RCTs), non-randomised controlled trials (non-RCTs), before and after studies, and interrupted time-series studies. In addition, analytical observational studies, including prospective and retrospective cohort studies, case–control studies, and analytical cross-sectional studies, were considered for inclusion. This review also examined descriptive observational study designs, including case series, individual case reports, and descriptive cross-sectional studies. Qualitative studies were also considered, which focus on qualitative data, including, but not limited to, designs such as phenomenology, grounded theory, ethnography, qualitative description, action research, and feminist research. Additionally, systematic reviews that met the inclusion criteria were also considered, depending on the research question. Furthermore, text and opinion papers were also considered for inclusion in this scoping review. Source exclusions comprised of experience reports, ongoing trials, incomplete articles, book chapters, editorials, and proceedings.

The review included studies published in the English language due to a dearth of resources for the translation expenses and time required. Although considered a limitation in terms of effect and conclusions, a recent systematic review conducted by Dobrescu et al. (27) revealed that limiting studies solely to English had little impact. Studies that were published between 23 August 2013 and 15 April 2024 were included to ensure that the review assessed current practice. The initial search date was 23 August 2023, and a 10-year search limit was deemed robust and credible for a scoping review. The search was conducted with the assistance of a member of the academic librarian staff of the university (HH).

An initial limited search of MEDLINE and CINAHL was conducted in order to identify articles on the topic. The text words incorporated in the titles and abstracts of relevant articles, along with the index terms used to describe the articles, were used to devise a full search strategy. The search strategy included all identified keywords and index terms and was then adapted for each included database. The searched databases include Scopus, ProQuest, CINAHL, and Medline. Sources for unpublished studies / grey literature searched included the World Health Organization’s Registry Network, ClinicalTrials.gov, OpenGrey, Google Scholar, and the library catalogue. Finally, the reference list comprising all the sources of evidence was screened for additional research. The subject headings and keywords used to search these databases are listed in Table 1.

**Table 1:** Search terms.

Subject heading	Keyword
Nurse	Nurs* Nurs* led
Diabetes	Type 2 diabetes Non-communicable diseases Diabetes mellitus Diabetic foot Diabetic ulcer Diabetic complications
Foot prevention	Foot ulcer prevention Foot management Self-care
Education programmes	Education interventions Education and support
Sub-Saharan Africa	Uganda East Africa Tanzania Low- or middle-income countries (LMIC)
Self-management	Support programmes

### Study selection process

Following the search, all identified citations (*n* = 1870) were collated and uploaded into EndNote 21 and all duplicates removed (*n* = 16). Three independent reviewers screened the titles and abstracts of 1845 citations for assessment against the PCC inclusion criteria for the review. Sources potentially relevant were retrieved in full (*n* = 23). The full text of selected citations was assessed in detail against the inclusion criteria by five independent reviewers. The reasons for the exclusion of sources (*n* = 9) of evidence at full text that did not meet the inclusion criteria were as follows: two studies on the impact of a diabetes self-management educational programme were not nurse-led (28, 29). One study reported on feedback from patients with type 2 diabetes on how to adapt a DSME programme, rather than an evaluation of it (30). Another study evaluated the experiences of the educators but not the impact on the participants (31). One study was a study protocol (32), and another was a process study (33). According to a mixed-method study by Lamptey et al. (8), DSME interventions were delivered by nurses, doctors, and/or nutritionists in two urban low-resource primary settings. The third in the study, findings of an RCT by Diriba et al. (34–36), was excluded because it reported on the perceived support status of people with T2DM and support behaviors in family caregivers. We found one study on nurse-led diabetes foot screening and education by Hall et al. (18), but this study was excluded because the researchers were examining if the nursing staff documented their assessments and patient education on at least 25% of visits only and not the impact on reducing foot ulceration.

**Figure 1:**
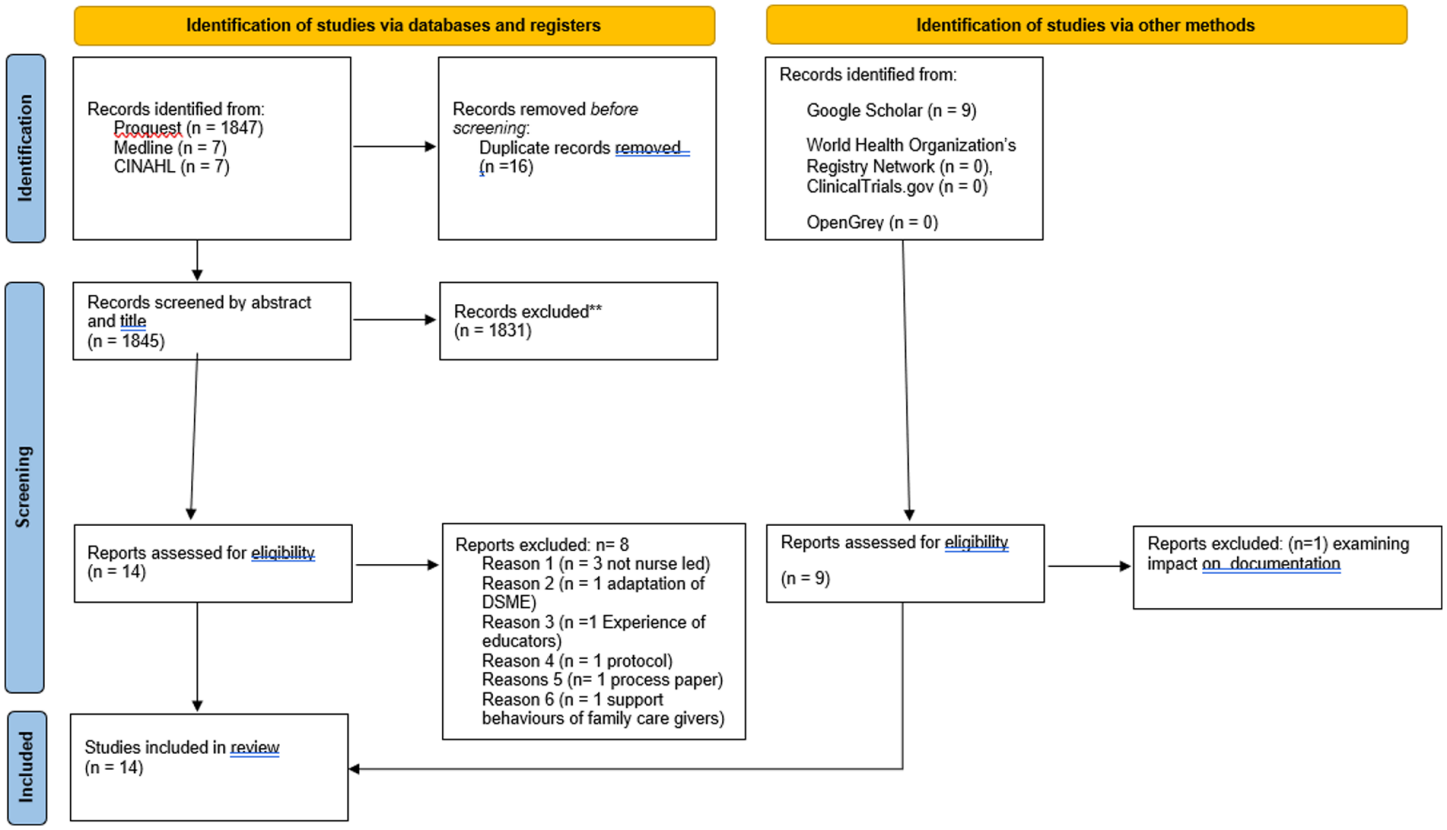
PRISMA flow chart.

Any disagreements that arose among the reviewers at any stage of the selection process were resolved through a collaborative discussion among them. This involved a discussion regarding the inclusion of three studies (8, 37, 38), which were led mainly by nurses, but doctors occasionally facilitated the sessions. Since they were mainly nurse-led, it was agreed that they should be included in the results. The complete report of the results of the search and the study inclusion process is presented in a Preferred Reporting Items for Systematic Reviews and Meta-Analyses extension for Scoping Review (PRISMA-ScR) flow diagram (26) (see Figure 1).

### Data extraction

Data was extracted from the included papers by six independent reviewers using a data extraction tool developed by the research team. The extracted data included specific details about the participants, concept, context, study methods, and key findings relevant to the review question(s) (see Table 2). The objective was to identify and map the level of evidence for nurse-led diabetic foot prevention programmes and education programmes for type-2 diabetes in SSA. The countries in which the studies were conducted, the types of programmes and interventions implemented, including the characteristics of the programmes (place of delivery, strategies and theories employed, content, frequency, duration, use of supplementary material), the participants (sample size, inclusion, exclusion), the outcomes measured and when they were measured, the facilitators and/or barriers that have been reported pertaining to the success and/or failures of the services/programmes, and the impact of the services/programmes on patients and staff.

**Table 2:** Data extraction.

Author(s)	Year	Participants	Concept	Context	Study methods	Key findings relevant to review questions
Bett	2019	Total sample size of 143 patients was split into 2 groups. T2D with HbA1c over normal limits. Able to read, write, and speak English. Aged 25–75 years and Black African.	The purpose was to determine if a structured diabetes education intervention for patients in an urban–rural hospital would improve patients’ diabetes management.	An urban–rural hospital in Eldoret, Kenya. The experimental group was given lessons on a structured diabetes education once every week for three weeks and then followed up for three months as part of a four- and half-month programme. 3 × 120-min classes over 1 week.	A non-randomised experimental design. All the participants were given a diabetic knowledge test (DKT) and self-efficacy test at the beginning and at the end of the project. In addition, each participant’s HbA1c was collected before and after the intervention.	At the end of the three months’ intervention, 123 out of 143 (86%) had completed the project (60 control and 63 experimental). The results showed that the experimental group had significantly reduced their levels of HbA1c compared to the control group. Also, the experimental group improved their diabetic knowledge and self-efficacy significantly compared to the control group.
Brady et al.	2021	98 participants and 12 trained health workers Patients T2D over 18 years. Exclusion Criteria: severe and enduring mental health problems; not primarily responsible for their own care; not provide informed consent; not able to participate in activities in a group setting or currently participating in another study.	Trial culturally and contextually adapted version of UK approach (EXTEND) for T2D.	2 urban settings; a public health centre in Malawi and (private OPD) Mozambique. 2 × 3-h sessions Delivered by 5 nurses and 1 medical student across 2 sites.	Single Group Feasibility Study No control group—unethical to exclude people. Mixed methods evaluation. Quantitative: Physiological data HbA1c, total cholesterol, low-density cholesterol, triglycerides, BP, HR, BMI (weight and height), Health, and Wellbeing questionnaires. Qualitative: interviews and focus groups.	Positive biomedical (HbA1c and BP) and psychological outcomes but unable to test cost-effectiveness in resource-poor settings. Attending a self-management program can bring about positive behavior change and improved emotional well-being.
Diriba et al.	2023	76 participant– caregiver dyads (people with T2D and one of their nominated family caregivers) Aged 18 years +; lived in 2 selected Kebeles; primary family caregiver for support; taking insulin and/or oral hypoglycaemic agents. Excluded pregnant; a physical disability to perform self-management practice; unable to understand Afaan Oromoo.	To examine the preliminary effects of a culturally tailored, family supported, community-based diabetes self-management education and support (DSMES) programme for people with type 2 diabetes on glycosylated haemoglobulin (HbA1c), blood pressure, body mass index, and lipid profiles.	Western Ethiopia, 3 community centres. Delivered 6 × 2 h face-to-face culturally tailored DSMES sessions on top of usual care, with family support attendance. Included an education package, 2 videos (insulin administration and foot care), and fliers for each session	Two-arm pilot randomised controlled trial (RCT). Primary outcomes reported in the study were HbA1c 2 months after intervention. Secondary outcomes reported were blood pressure, BMI, total cholesterol, LDL, HDL, and triglycerides.	Significant improvement in HbA1c with large effect size (β = −1.667, *p* < 0.001, d = −0.81) and triglycerides with medium effect size (d = −0.50). HbA1c in the intervention group was decreased by 12 mmol/mol (1.1%). Although nonsignificant, the DSMES also had small to moderate effects (d = −0.123 to 0.34) on blood pressure, body mass index, total cholesterol, and low-density and high-density lipoproteins when compared with usual care.
Diriba et al.	2024	76 participant– caregiver dyads (people with T2D and one of their nominated family caregivers) Aged 18 years +; lived in 2 selected Kebeles; primary family caregiver for support; taking insulin and/or oral hypoglycaemic agents. Excluded pregnant; a physical disability to perform self-management practice; unable to understand Afaan Oromoo.	To examine the preliminary effects of a culturally tailored, family supported, community-based diabetes self-management education and support (DSMES) programme for people with type 2 diabetes on Diabetes Self-Management Behavior and Quality of Life	Western Ethiopia, 3 community centres. Delivered 6 × 2 h face-to-face culturally tailored DSMES sessions on top of usual care, with family support attendance. Included an education package, 2 videos (insulin administration and foot care), and fliers for each session	Two-arm pilot randomised controlled trial (RCT). This study reports on Diabetes Self-Management Behavior and Quality of Life Summary of Diabetes Self-care Activities-Expanded (SDSCA) and 34-item diabetes quality of life-Afaan Oromoo (DQOL-AO) scale	The DSMES programme outperformed usual care, with large effect sizes at T1 (β = 1.429, *p* < 0.001, d = 1.47) and T2 (*β* = 2.216, *p* < 0.001, d = 1.81), in the overall DSM practice. Statistically significant differences in the changes in all DSM practice subdomains over the study period between the two groups, with the DSMES programme having outperformed usual care, with medium-to-large effect sizes at T1 and T2. The DSMES programme outperformed the usual care approach, with large effect sizes (β = −0.833, *p* < 0.001, d = −1.48), in the overall QOL. The GEE results showed statistically significant differences in the changes in all QOL subdomains over the study period between the two groups, with the DSMES programme outweighing usual care, with medium-to-large effect sizes
Emmanuel et al.	2017	20 diabetics from medical outpatients (12 men, 8 women; age range 25–65 years)	Effect of Nurse-led Training on Self-management of Diabetes among Diabetic Patients Attending Medical Outpatient Clinic in General Hospital Odan, Lagos State, Nigeria	2-h training delivered by nurses to the participants over a clinic in 1 week.	One group pre-test, post-test quasi-experimental study was adopted. Pre- and post-intervention questionnaire (developed by the research team).	There was 70% increase in knowledge regarding diabetes self-management among participants post-intervention. There was a 45% increase in practice regarding diabetes self-management among participants post-intervention. The result showed a significant difference in the effect of nurse-led training on knowledge regarding self-management among diabetic patients pre- and post-intervention with a mean difference in knowledge score of 14.2 (*p* = 0.000). Result also showed significant difference in effect of nurse-led training on practice regarding self-management among diabetic patients pre- and post-intervention with a mean difference in practice score of 1.05 (*p* = 0.000).
Essien et al.	2017	Adult T1 or T2 diabetics, HbA1c levels >8.5%, 30 days prior to randomisation, able to engage in moderate exercise without issue and free of any eye disease that would otherwise limit their ability to read. N = 59 interventions (49 were T2) N = 51 control	To evaluate whether an intensive and systematic DSME programme, using structured guidelines, improved glycaemic control compared to the existing *ad hoc* patient education programme.	One teaching hospital in Nigeria. 3 doctors and 3 nurses trained as certified instructors. Delivered 12 structured teaching sessions x 2 h each, fortnightly over a 6-month period. The initial 6 sessions were delivered by 3 doctors, whereas the final 6 sessions were delivered by 3 nurses. Included interaction, videos, and leaflets to take home. Location was away from the clinic. 6–8 participants in a session. Mobile phone reminder messages for the next session were sent to participants.	Unblinded, parallel-group, individually randomised controlled trial. Primary outcome was HbA1c (%), recorded six months after randomisation (± at most five days).	Intensive group participants having HbA1c outcomes on average − 1.8 (95% CI 2.4 to −1.2) percentage points lower than conventional group participants.
Hailu et al.	2018	Adult patients with type 2 diabetes. 30 years +, overweight or obese, taking oral hypoglycaemics or insulin. Excluded T1DM, gestational diabetes, or a severe mental or physical incapability, terminally ill *n* = 78 intervention and = 64 control	Develop and test the effectiveness of a multifaceted, nurse-led DSME program for improving diabetes knowledge, self-care activities, and self-efficacy.	Medical centre, Jimma City in Ethiopia. Delivered by 2 nurses who received 16-h training. 6 educational sessions, 1.5 h on average. Also included an educational handbook and fliers adapted to the local context; and interactive discussions with peers and take-home activities.	Controlled clinical trial, before-and-after, two-group intervention study. Primary outcome reported was a change in the proportion of people with target glycated haemoglobin (HbA1c ≤7%).	Mean HbA1c was significantly reduced by 2.88% within the intervention group and by 2.57% within the comparison group. However, the change in the proportion of participants with target HbA1c and end-line mean HbA1c difference between the groups was not significant. Adjusted end-line fasting blood sugar (FBS), systolic blood pressure (SBP), and diastolic blood pressure (DBP) were significantly lower in the intervention group, by 27 ± 9 mg/dL, 12 ± 3, and 8 ± 2 mmHg, respectively.
Hailu et al.	2019	Adult patients with type 2 diabetes. 30 years +, overweight or obese, taking oral hypoglycaemics or insulin. Excluded T1DM, gestational diabetes, or a severe mental or physical incapability, terminally ill *n* = 78 intervention and *n* = 64 control	Develop and test the effectiveness of a multifaceted, nurse-led DSME program for improving diabetes knowledge, self-care activities, and self-efficacy.	Medical centre, Jimma City in Ethiopia. Delivered by 2 nurses who received 16 h training. Delivered 6 educational sessions, 1.5 h on average; handed out a colourful, well-illustrated educational handbook and fliers adapted to the local context; and provided extensive and interactive discussions with peers and take-home activities. 8–12 participants per group.	Controlled clinical trial, before-and-after, two-group intervention study. Secondary outcomes reported: Diabetes knowledge Diabetes self-care behaviors Diabetes self-efficacy	Intervention group (a) had a greater mean diabetes knowledge score, 11.33 out of 20, compared to that of the comparison group, 10.61 out of 20 (*p* = 0.050). (b) followed general dietary recommendations for 5.06 days per week, which is statistically significantly greater than the 4.44 days reported by the comparison group (*p* = 0.027). (c) performed footcare for a mean of 5.80 days per week, compared to 5.26 days for the comparison group (*p* = 0.009). No statistical significance in exercise, BGM, self-efficacy, smoking, alcohol consumption, and khat chewing.
Lamptey et al.	2023	Adults aged over 18 years with T2 diabetes Excluded those with CKI and Sickle Cell. Intervention *n* = 79 + *n* = 80 control.	Compare structured EXTEND programmes to usual care in LMICs.	Conducted at two hospitals (WGMH and KBTH) in Accra, Ghana. 5 community health nurses and one medical officer delivered a structured and culturally adapted (Ghana) EXTEND programme. One 6-h session delivered by two educators (not direct care providers) within 2 weeks of randomisation 6 to 10 participants within 2 weeks of randomisation.	A multicentre, parallel-group, single-blind randomised controlled trial. Primary outcome was change in HbA1c after 3-month follow-up. Secondary outcomes were changes in clinical, psychological, and self-care variables. Specifically, the clinical outcomes were change in weight, waist circumference, and blood pressure, respectively; the psychological outcomes were changes in diabetes-related distress scores and WHO quality of life scores, respectively; and the self-care outcome was change in diabetes self-care activities (SDSCA) scores.	HbA1c decreased within both groups: −0·9% in the intervention group and − 0·3% in the control group. The decrease was greater in the intervention group but was not significant. Insufficient evidence that the intervention had an effect on any of the secondary outcomes except for an improvement in physical health. Differences were significant for self-care activities, namely foot care, exercise, and diet.
Lubega et al.	2023	20 adult participants are not clear if T2 or T1 diabetic. 12 were female and 8 were male, with age ranging from 38 to 65 years and residing within a radius of 1 km from the health club’s meeting point. Majority (16) had lived with diabetes for 5 or more years, whereas four participants had been diagnosed in the last 2 years	Explored the role of nurse-led community-based health clubs in promoting patients’ health education for diabetes self-care management towards lifestyle modification, adherence to treatment, reduced risk factors, and improved dietary behaviors and glycaemic control among patients	Two community-based health clubs in two urban settings of Nansana and Mende villages along Kampala–Hoima Highway in Wakiso District in Uganda. 8 weeks of 2–3 h of meetings on each Sunday. 2 final-year nursing students (BSc)	A cross-sectional qualitative study. 3 focus groups of 6–8 participants.	Three major themes on the role of community health clubs in promoting health education for diabetes self-care management were developed. These include promoting the sharing of experiences, improving awareness of healthy living practices, and offering sufficient patient–health worker interaction time.
Okafor et al.	2021	382 patients with T2 diabetes Experimental group *n* = 198	The effect of an ACP nurse-led educational intervention program on the self-management practices of individuals with Type 2 Diabetes Mellitus in the South East, Nigeria	2 hospitals experimental and 2 control. Delivered a 9-week programme and gave out booklets to participants to take home. Follow up phone calls and meetings every 2 weeks to emphasise self-care practices. After 6 months and end of post-test educated the control group and gave booklet. Both groups and carers received psychoeducation	Multi-centre quasi-experimental design Summary of Diabetes Self Care Activities (SDSCA) Scale. The SDSCA instrument assessed self-care practice in areas such as exercise, diet, and self-blood glucose monitoring (SBGM), footcare and medication, which are areas of daily self-care activities for individuals with DM.	No significant difference was observed in self-management practices between the experimental and control groups prior to intervention (χ2 = 0.180–3.351, *p* ˃ 0.05). However, 6-months after intervention, significantly higher mean rank was observed in the intervention group in the diet domain (*χ* 2 = 23.817, *p* = 0.001), exercise (χ2 = 11.545, p = 0.003) and in foot-care (χ 2 = 168.217, *p* = 0.001) indicating the effectiveness of educational intervention.
Okafor et al.	2023	382 T2 Diabetics Experimental Group *n* = 198	The effect of an ACP nurse-led educational intervention program on the quality of life of individuals with Type 2 Diabetes Mellitus in the South East, Nigeria in comparison to usual care.	2 hospitals experimental and 2 control. Delivered a 9-week programme and gave out booklets to participants to take home. Follow up on phone calls and meetings every 2 weeks to emphasise self-care practices. After 6 months and the end of the post-test, educated the control group and gave booklets. Both groups and carers received psychoeducation	Multi-centre quasi-experimental design – HRQoL questionnaires. Primary outcome no difference in HRQoL between groups.	No significant difference in experimental and control groups in sex, gender, and age. HRQoL higher pre-test in control. 6 months later, experimented significantly higher HRQoL scores. Educational intervention for people living with diabetes will be helpful in the non-medical management of DM.
Okafor et al.	2023 b	382 T2 Diabetics Experimental Group *n* = 198	The effect of an ACP nurse-led educational intervention program on the self-efficacy of individuals with Type 2 Diabetes Mellitus in the South East, Nigeria in comparison to usual care.	2 hospitals experimental and 2 control. Delivered a 9-week programme and gave out booklets to participants to take home. Follow up on phone calls and meetings every 2 weeks to emphasise self-care practices. After 6 months and the end of the post-test educated the control group and gave booklets. Both groups and carers received psychoeducation	Multi-centre quasi-experimental design: Stanford Chronic Disease Self-Efficacy Scale pre- and 6 months’ post-intervention.	There was no statistically significant difference between the two groups before intervention. However, after 6 months of intervention, a significant proportion of participants’ scores in the intervention group moved from low to either moderate or high SE in almost all the SE domains (*p* < 0.05).
Tamiru et al.	2023	278 participants completed (*n* = 153 intervention) adults aged over 18 years. Patients with severe cognitive or physical impairment and terminally ill people with serious diseases, such as severe cardiovascular and cerebrovascular diseases, severe kidney disease, cancer, and visual impairment due to complications of type II DM, were excluded.	To assess the effect of DSME on self-care knowledge and behavior among adult people with type II diabetes attending diabetic follow-up clinics in selected hospitals.	Conducted in 4 hospitals in the Ilu Abbabor and Buno Bedelle Zones, southwest Ethiopia. Nurse-led DSME to 8 to 10 patients every two weeks for one and a half hours for six consecutive months.	Quasi-experimental study design. The Diabetes Knowledge Test (DKT) was used to assess diabetes self-care knowledge. Self-care behavior was assessed using the Summary of Diabetes Self-care Activities.	No significant difference in all of the outcomes before intervention between control and intervention groups; however, there was a statistically significant higher mean score difference in self-care knowledge and self-care behavior after the delivery of DSME (*p* < 0.05)

The critical appraisal of individual sources of evidence is generally not required for scoping reviews. However, the reviewers sought to assess the evidence for its relevance, reliability, validity, and applicability. Some of the limitations of the papers included in the scoping review comprise small sample sizes of study participants in some of the studies (37, 39–43). In some of the studies, a loss of follow-up of participants was reported, which may potentially impact the estimated DSME effect (44). All of the studies only evaluated the short-term impact of the DSME; therefore, the long-term effect on glycaemic control and other outcomes is unknown. Some of the studies only reported limited outcome measures, with three studies reporting findings from the same research in other papers (34–36, 41–43, 45–47), which may fragment the findings that could have been derived if the data were presented as a whole (48). In some of the RCTs, patients and providers were not blinded to the intervention or to the results of the outcomes, which could produce bias. The study population was not heterogenous in all the projects, and this may affect the generalisability of the findings (38, 46, 47). The settings in which certain studies were conducted are also limitations, such as those conducted in a single location, which may impact generalisability (37–39, 46, 47, 49). There was also an absence of a control group in certain studies, thus it cannot be ruled out that the observed changes were due to chance or a maturation effect (37, 41–43). Some of the focus groups and interviews in the studies used an interpreter, which could have introduced bias.

### Data analysis and presentation

This review included 14 articles. Table 2 summarises the primary characteristics. Notably, multiple papers originate from the same three studies (34–36, 41–43, 45–47), resulting in a total of 10 distinct studies.

Relevant information from the papers was extracted in a systematic way in order to report on the objectives of this review. To report on nurse-led foot prevention programmes and DSME interventions, the types of DSME, and their impact. The factors that facilitated and hindered the successful implementation of nurse-led diabetes self-management education programmes were synthesised narratively using a descriptive qualitative content analysis approach (25). The analysis of the data items was an iterative process conducted by all members of the team. This involved quantifying text and creating frequency tables of quantitative data (50). Due to a lack of literature, an inductive approach was used. Initially, the authors familiarised themselves with the data, then used open coding to list initial thoughts and create categories. These were reviewed by all team members at frequent meetings and discussions regarding the inclusion of papers that came from the same study to ensure that work was not double counted.

#### To report on nurse-led diabetic foot prevention and education programmes for patients with type 2 diabetes in Sub-Saharan Africa

The scoping review made a crucial observation that no programmes or interventions were exclusively dedicated to nurse-led prevention of diabetic foot complications. However, all nurse-led initiatives (*n* = 10) were focussed on delivering education on self-managing diabetes. The studies incorporated various educational, behavioral elements, and/or lifestyle changes related to diet and exercise and were primarily targeted at individuals with type 2 diabetes. However, two studies (39, 40) did not clarify the type of diabetes the participants presented with. The studies compared the outcomes of the participants following their DSME participation with standard care. The outcomes included clinical and non-clinical aspects. The review encompassed studies where DSME interventions were led by nurses. However, in three instances (8, 37, 38), the primary leadership was provided by nurses with doctors offering support.

Across the studies, the total number of participants in the DSME interventions was 797.

The SSA countries where the studies were conducted included Ethiopia (3), Nigeria (3), Kenya (1), Mozambique (1), Malawi (1), Uganda (1), and Ghana (1). One study (37) was conducted across two countries, Malawi and Mozambique. The research methods used to collect data across the studies varied, with the majority using a quantitative methodology as part of mixed methods or a standalone approach. One study (40) used a qualitative approach using focus group interviews. Out of the remaining nine studies, four were randomised controlled studies (8, 34–36, 38, 45–47), and five studies were quasi-experimental (37, 39, 41–44, 49).

The frequency and duration of the educational intervention and follow-up period varied considerably across the studies, although none extended beyond six months (Table 2). Total hours of educational input varied from 2 to 24 h. Session lengths ranged from 90 min to 6 h, with the majority lasting 2–3 h. The time interval between training inputs was often unclear. Studies of longer durations tended to have fortnightly sessions. Follow-up took place after six months of the intervention commencement in 50% of cases and after shorter periods in the remaining studies.

As illustrated in Table 2, the nurse-led DSME interventions predominantly adopted a behavior-change strategy. This strategy was designed to enhance diabetes knowledge, self-efficacy, and self-management skills through various methods. These included non-didactic teaching methods, interactive discussions, goal-setting activities, support provision, caregiver/family involvement, the utilisation of community health clubs, and psychoeducation for participants and their families in order to enhance diabetes outcomes. All studies included education on diabetes and related factors, as well as self-management of the condition. In addition, the interventions provided patients with educational support materials, such as handbooks, booklets, flyers, and pamphlets. Additionally, the studies covered expenses for patients, such as travel and refreshments. Studies reported multiple follow-up strategies, such as phone calls, WhatsApp group support, and appointment reminders.

Five studies (34–37, 40, 45, 49, 51) made references to theoretical frameworks as the foundation of the DSME, such as dual process theory (52), self-efficacy (53), social learning theory (54), empowerment theory (55), social cognitive theory (54), Leventhal’s common-sense approach (56), health belief model (57), and chronic care model (58). The application of these theories was described in some studies, whereas it was not clear in others. For instance, Diriba et al. (34–36) identified the social cognitive theory as the foundational theory for the DSME. They incorporated family members into the educational sessions and engaged them in providing continuous support for the patients.

Four studies (34–37, 40, 46) have reported using service users or patient and public involvement (PPI) in the design and development of the DSME. The EXTEND programme by Brady et al. (37), was produced in collaboration with PPI, who were involved in choosing the content of the DSME that applied to their context. Patients actively participated in the project design by suggesting areas of focus for health education discussions and assigning the community clubs close to patients’ homes in the study by Lubega et al. (40). In Diriba’s (35) study, patients were involved in validating the educational handbook, and Hailu et al. (46) consulted 27 patients with T2DM for context-specific expertise to develop the DSME programme.

Group education (with a range of 8–12 participants) was the key strategy used across all the studies. All of the included studies used a face-to-face format. The interventions were either facility/hospital or community-based, with six studies being conducted at tertiary hospitals and four being community-based.

#### To examine whether there are different types of nurse-led diabetic foot prevention services and structured educational programmes

Two studies (8, 37) used a structured DSME called EXTEND (EXTending availability of self-management structured education programmes for people with type 2 Diabetes in low- or middle-income countries). This was a cultural and contextual adaptation of a DSME called DESMOND, which was originally developed in the United Kingdom and meets international criteria for DSME. It has previously been demonstrated to be effective and cost-effective for individuals with T2DM. EXTEND was piloted in Malawi and Mozambique, as reported by Brady et al. (37), and was culturally adapted in Ghana by Lamptey et al. (8). Local research teams invited a PPI group to attend a 2-day session in which the contents of the UK DSME were contextualised to meet local needs.

The other eight studies used locally developed DSME programmes that were based on various sources. The DSME interventions used were primarily structured to meet national or international standards. These include the Diabetes Education Training Manual for Sub-Saharan Africa (59), recommendations from the American Diabetes Association (60), and the seven key behaviors of DSME by the American Association of Diabetes Educators (61), the European Association of Study of Diabetes (EASD) (62), the International Diabetes Federation (59), and the UK’s National Institute of Health and Care Excellence (63) standards. Among the studies where DSME was developed locally, three of them (34–36, 40, 46) involved PPI in the design and content selection of DSME.

#### Enablers and barriers of nurse-led DSME interventions

The studies have identified a range of factors (enablers and barriers) that impact both the acquisition of new knowledge and the potential for that new knowledge to influence active behavior change in the participants of the DSME’s. These issues can be grouped into a number of themes, including program design (content and pedagogy), operational issues impacting workshop attendance, and ‘structural’ (socio-economic) issues impacting users’ abilities to make changes to their lives.

#### Programme design

##### Content

All the studies suggested the importance of programmes taking a holistic approach and, either explicitly or implicitly, addressing all seven self-care behaviors, including nutrition, exercise, monitoring of blood sugar, compliance with medications, problem-solving and coping skills, and risk-reduction behaviors (64). However, a surprising finding was that integration of footcare education was rare.

##### Communication

The reviewed studies highlighted that effective and contextually appropriate communication, both in the theoretical aspects of the DSME sessions and in the handouts/leaflets to take home, which was attentive to linguistic diversity and literacy competence, improved participant learning. This was further enhanced when the DSME was co-produced with service users. Coproduction was found to be a valuable strategy because it ensures and improves chances that both the pedagogic style and content of the DSME are attuned to the experiences and competencies of the users to whom it is being delivered. Furthermore, co-production of the DSME also improves the management of any tensions between new (scientific) knowledge and existing cultural beliefs and practices. Finally, by deploying a variety of learning strategies, including demonstrations, group work, videos, exercises, and experiential/knowledge sharing, where diverse communication styles are utilised, participant learning is improved.

##### Delivery mechanism

Across the studies, those who utilised face-to-face delivery in small groups (8–12), in sessions of 1.5–2-h duration and spread across a number of sessions, achieved optimal learning outcomes among the participants. The DSME’s who provided contextually appropriate supporting materials to take home for family and users were welcomed by the participants.

#### User participation

On analysis of the studies, it was found that user participation was impacted by the costs of travel and the timing and time commitment involved. The DSME’s that gave support with transport costs improved attendance and/or those that timed the programme to coincide with other visits (to collect medications, etc.).

The stock-out of medications at local facilities discouraged attendance, which both impeded the participants ability to combine two journeys but also to comply with the advice on medication management.

It was noted that the timing of the DSME sessions should include the avoidance of public holidays and take into account the impact of weather (especially during rainy seasons), as this affects attendance and attrition.

DSMEs that held clinics as locally as possible to patients or close to where they—or where they already attend—were able to optimise attendance and participation.

#### Structural factors

Various external factors were discovered throughout the studies to have an impact on the participants’ abilities to apply new knowledge (which may also impact their receptiveness to learning). It also included the costs associated with changing their diet or footwear. Where participants had more complex health needs (comorbidities) as well as T2DM, it was noted that they may felt they had to prioritise those that impact them most tangibly (e.g., hypertension).

Across the studies, it was noted that participants valued opportunities for on-going support (through telephone/WhatsApp etc.).

Two interesting observations that emerged from the analysis of the data that do not address the objectives of the review, but the research team felt were of interest: categorising health workers and optimising the evidence base.

#### Health workers

Lamptey et al. (51) and Bett (49) identified in their findings that those developing DSME’s in SSA should ensure an appropriate amount of time is dedicated and provided to develop contextualised materials, etc., and deliver the program. In their study, the projected time was 12 weeks, when it took 26.

Despite the emphasis on training nurses to deliver DSME’s, there is value in partnering with other members of the multi-disciplinary team (both in terms of scientific knowledge and pedagogy).

##### Optimising the evidence base (why things work/do not work)

The included studies in this scoping review referred to the theories that supported the design and delivery of the DSME’s, but the factors that influenced and determined the selection of the theories were not examined consistently by all. The aim of referring to this is not to compare theories, but rather to understand how the theories could provide a systematic method for the development and delivery of DSME’s in SSA in the future. Without argument for their usefulness, their application to future studies is limited.

#### To assess the impact of the services/programmes on patients and staff

##### Patient impact

The fourth objective of this scoping review was to examine the impact of the nurse-led DSMEs on the patients who participated in them. According to the standards of DSME set out by the ADA, the outcome data can include clinical (HbA1c or glycated haemoglobin or A1c, blood pressure, weight, and so on), psychosocial and behavioral outcomes (healthy eating, taking medications, being active, etc.), patient-reported outcomes (health-related quality of life, self-efficacy, diabetes distress, patient satisfaction), and patient-generated health data (blood glucose trends, steps taken, sleep, etc.). The review indicated that there were four outcome themes: knowledge outcomes, behavior outcomes, quality of life outcomes, and clinical outcomes.

From across the studies, it appears that the Michigan Diabetes Knowledge Test is the most used tool for assessing diabetes knowledge pre- and post-intervention (Table 3). However, diabetes self-efficacy testing was measured by using a modified Stanford diabetes questionnaire. Studies such as Hailu et al. (47) and Diriba et al. (45) have reported that these tools were translated into the local language and modified to include or exclude certain questions to meet the context. The summary of diabetes self-care activities (SDSCA) was widely used to assess the behavioral outcomes of the DSME, whereas the psychological impact of the DSME was assessed using several tools, such as Quality of Life questionnaires and SF-36 questionnaires.

**Table 3:** Outcome measures.

Author, year	Outcome measures			
	Knowledge outcomes/tools	Behavior outcomes/tools	Quality of life outcomes/tools	Clinical outcomes
Bett (49)	Self-efficacy modified (SCDES) Diabetes knowledge modified (DKT)	–	–	HbA1c
Brady et al. (37)	Self-efficacy (SF-20)	Behavior changes qualitative interview	Quality of life using the WHO (FIVE) wellbeing index, problem areas in diabetes (PAID score, and PHQ-9)	HbA1C, FBS, BP, lipids, BMI, weight, waist circumference
Diriba et al. (34–36)	–	–	–	HbA1c, BP, lipids, BMI, weight
Diriba et al. (45)	–	Self-management behavior, using a SDSCA	Quality of life using a 34-item diabetes quality of life measure	–
Essien et al. (69)	–	–	–	HbA1c
Hailu et al. (46)	–	–	–	HbA1c, FBS, BP, BMI, waist circumference, waist to hip ratio
Hailu et al. (47)	Diabetes knowledge (DKS) Self-efficacy (SCDES)	Self-care behavior (SDSCA)	–	–
Lamptey et al. (8)	–	Self-care outcomes (SDSCA)	Changes in diabetes-related distress score (PAID-5) and WHO QOL	HbA1c, BP, waist circumference
Lubega et al. (40)	Diabetes knowledge: qualitative study using structured interview	–	–	–
Okafor et al. (41)	–	Self-management practices (SDSCA)	–	–
Okafor et al. (42)	–	–	Quality of life SF – 36	–
Okafor et al. (43)	Self-efficacy (SCDES)			
Olajide et al. (82)	Diabetes knowledge no tool mentioned	Self-management No tool		
Tamiru et al. (44)	Diabetes self-care knowledge (DKT)	Self-care behavior (SDSCA)		

*Stanford Chronic Disease Self-Efficacy Scale (SCDES), Simplified Michigan Diabetes Knowledge Scale (DKS), Quality of life using short form 36-QOL-SF-36, Quality of life-WHO QOL brief instrument, Self-management practices using Summary of Diabetes Self Care Activities (SDSCA), Diabetes-related distress with, the problem areas in diabetes-5 (PAID-5), 20 item short form survey scale, SF-20.

It was interesting to observe that the clinical outcomes measured were varied across the studies, with the exception of the HbA1c, which was used across all the studies that evaluated the clinical outcomes. The common outcomes were glycated haemoglobin (HbA1c), systolic and diastolic blood pressures, weight, body mass index (BMI), waist circumference, waist-to-hip ratio, and lipid profile (Table 4).

**Table 4:** reported clinical outcomes of the DSME’s.

Author, year	HbA1c	FBS	BP	Lipids	BMI	Weight	Waist circumference	Waist to hip ratio
Bett (49)	√	×	×	×	×	×	×	×
Brady et al. (37)	√	√	√	√	√	√	√	×
Diriba et al. (34–36)	√	×	√	√	√	√	×	×
Essien et al. (38)	√	×	×	×	×	×	×	×
Hailu et al. (46)	√	√	√	×	√	×	√	√
Lamptey et al. (8)	√	×	√	×	×	×	√	×

#### Clinical outcomes

Across the ten studies, only six reported on clinical outcomes (8, 34–38, 46, 49), which is summarised in Table 4.

##### Glycated haemoglobin (HbA1c)

HbA1c was the commonly used clinical outcome measure to assess the impact of nurse-led DSME programmes. The HbA1c is a blood test that displays your average blood glucose levels over the past 2–3 months. Six studies have reported clinically significant improvements in HbA1c levels immediately following the DSME within the intervention group. Notably, four of these studies (34–38, 49) also demonstrated statistical significance in HbA1c reduction.

##### Fasting blood sugar

FBS was measured in two studies, and both showed some clinically relevant changes in the group after the DSME (37, 46). However, these changes were not statistically significant.

##### Blood pressure

Blood pressure appears to be the next commonly used clinical measure, assessed by four studies reporting clinical outcomes. Two studies (37, 46) demonstrated clinically and statistically significant improvements in blood pressure within the intervention group. One study (34–36) demonstrated a small to medium effect, whereas blood pressure remained unchanged in the intervention group in the study conducted by Lamptey et al. (8).

##### Lipid profile

Two studies (34–37) reported on the impact of lipid levels, including total cholesterol, LDL cholesterol, HDL cholesterol, and triglycerides. Brady et al. (37) demonstrated clinically and statistically significant reductions after DSME programmes, whereas Diriba et al. (34–36) found no difference between the intervention and control groups in total cholesterol or LDL cholesterol. However, a medium effect was observed in triglyceride levels.

##### Waist circumference and waist-to-hip ratio

Change in waist circumference was measured post-DSME in three studies (8, 37, 46), but no significant effect was noted. Additionally, Hailu et al. (46) measured the endline waist-to-hip ratio, showing no impact post-DSME.

##### Body mass index

Three studies (34–37, 46) measured BMI before and after DSME intervention and noted no difference after the intervention.

#### Learning, behavior, and quality of life outcomes

Non-clinical measures included in the outcome measures of the reviewed studies included diabetes knowledge, self-care behavior change, self-efficacy, and quality of life. Changes in diabetes knowledge after the education intervention, changes in the daily routine for managing diabetes, confidence in managing diabetes, and how diabetes affects overall happiness and well-being were measured using scales as shown in Table 3. The outcome measured by the individual studies included in the review is listed in Table 5.

**Table 5:** Learning, behavior, and quality of life outcomes.

Author, year	Diabetes knowledge	Behavioral outcomes	Self-efficacy	Quality of life
Bett (49)	√	×	√	×
Brady et al. (37)	×	√	√	√
Diriba et al. (45)	×	√	×	√
Hailu et al. (47)	√	√	√	×
Lamptey et al. (51)	×	√	×	√
Lubega et al. (40)	√	×	×	×
Okafor et al. (41–43)	×	√	√	√
Olajide et al. (82)	√	√	×	×
Tamiru et al. (44)	√	√	×	×

##### Diabetes knowledge

Five studies investigated the impact of DSME on diabetes knowledge, and all reported positive results after the nurse-led DSME interventions (40, 44, 47, 49, 82). The qualitative study by Lubega et al. (40) reported that participants acknowledging the role of the community club interactions and sessions in helping them get a better understanding of what they should do to have a healthy lifestyle.

##### Quality of life

Three studies (37, 42, 45) assessed quality of life and showed a positive impact on the quality of life of participants after the DSME intervention.

##### Self-efficacy scores

Four studies (37, 43, 47, 49) evaluated participants’ self-efficacy scores post-DSME. Three studies showed a positive impact, and one showed no impact (47).

##### Diabetes self-care behavior

Seven studies (8, 37, 41, 44, 45, 47, 82) investigated diabetes self-care behavior post DSME. Variation across studies and domains measured were noted. Notably, improvements in dietary choices were observed in several studies (8, 37, 41, 45, 47). Positive impact on lifestyle habits (such as exercise) was seen in some studies (8, 37, 45). Additionally, improvements in foot care behavior were noted by Hailu et al. (47), Lamptey et al. (8), and Okafor et al. (41).

### Staff impact

From across the 10 included studies, there is no data pertaining to the impact of the DSME’s had on staff.

## Discussion

The management of T2DM involves both continuous medical care and non-pharmacological self-care by the patient. SSA faces a rising burden of diabetes, but health care worker shortages, especially doctors, and limited resources pose challenges. This has led to increasing gaps in care delivery in LMICs (65). Recently, great emphasis has been placed on the role of nonpharmacological self-management in the care of patients with diabetes in recent years due to its close association with lifestyle and behavior modification. DSME plays a crucial role in facilitating this change. Published evidence favours innovative approaches such as task-shifting DSME design and delivery to nurses. Nurses, as the largest and most trusted health professional group, are uniquely positioned to inspire positive changes and transform health care delivery (66). A meta-analysis examining studies published until 2009 found that nurse-led diabetes self-management education (DSME) was associated with improved glycaemic control (67). However, the majority of the nurse-led DSME studies included in this meta-analysis were conducted in developed countries, and there was none from SSA, showing the gap in nurse-led DSME in SSA.

The studies included in this scoping review show that transferring DSME roles to nurses in SSA made the patient journey more efficient. Improvements in clinical, psychological, and behavioral outcomes illustrate this. Replicability, cost-effectiveness, and sustainability in a resource-stringent SSA context also benefitted from transferring DSME roles to nurses. Delegating tasks from physicians to nurses has been implemented across many services such as HIV/AIDS, tuberculosis, hypertension, diabetes, maternal and child health, etc. in Africa, as reported in a scoping review by Okoroafor and Christmals (68). This has proven to enhance access, coverage, and quality of care. Our review confirms the benefits created by nurse-led DSME in improving the way patients manage their diabetes. Nurse-led strategies in NCDs, such as DSME, therefore support the implementation of evidence-based system-level strategies to address the barriers caused by physician shortages. A nurse-led DSME is a crucial part of diabetes care, providing good glycaemic and metabolic control, which is vital for preventing long-term complications (69). This is particularly beneficial in low-resource settings, where it has been shown to positively impact diabetes knowledge, glycaemic control, and behavioral outcomes (44).

The review found limited nurse-led services specifically targeting diabetes foot assessment and prevention strategies. However, nurse-led DSME’s incorporated foot care skills into the 7 self-care behaviors. Despite resource constraints and a lack of experts like podiatrists in SSA (70), the review shows that educating patients on foot care as part of the nurse-led DSME has empowered them to enhance diabetes-related foot care skills. This finding underscores the value of investing in early DSME that can reduce the risks of complications, especially foot ulcers and lower limb amputations. A recent cross-sectional study in Uganda (71) and a systematic review by Stephanie et al. (72) showed that patients with diabetes in SSA had limited knowledge of diabetic complications, including diabetic foot and self-care practices, including foot care practices. In Africa, simple, repetitive foot care advice is crucial (70, 73), and integrating education to improve blood glucose, reduce cardiovascular risk, and include screening to prevent peripheral neuropathy and peripheral arterial disease into nurse-led DSME can further empower patients to prevent or delay devastating foot complications. Although two studies, Lamptey et al. (8) and Hailu et al. (46), included in this review, did not demonstrate statistically significant improvements in glycaemia, it is notable that foot care behavior significantly improved with the implementation of simple foot care advice as part of these nurse-led DSME interventions.

The scoping review delved into the different types of nurse-led DSME interventions used in SSA. It observed significant differences in the structure, design, duration of the education program, education strategies, follow-up duration, intensity of the intervention, use of theory to guide the interventions, use of guidelines to base the interventions, and the duration of educator training. Despite these variations, all studies employed a similar delivery approach: group-based education in an interactive, face-to-face format. The majority of the studies outlined the evidence-based guidelines used, and some references were made to the theories of learning and behavior change used in the DSME intervention. A systematic review conducted by Zhao et al. (6) evaluated the effectiveness of theory-based self-management interventions versus routine care for type 2 diabetes patients in randomised controlled trials (RCTs). The review found significant improvements in HbA1c levels, self-efficacy, and diabetes knowledge for theory-based interventions. The authors suggest that for theory-based interventions to have a greater impact, patients should play a more active and robust role, and the education team should receive training beyond the basic preparation for the self-management education program. Considering the variations noted across the included studies in terms of educator training, the review emphasises the gap in understanding and utilisation of theories of learning and behavior change pertinent to SSA as a foundation for DSME and the need for future research in this area.

This review investigated the effects of nurse-led DSME interventions on patients. It synthesised the impact on various outcome measures, including glycaemic control (e.g., HBA1c/FBG), cardiometabolic risk factors (e.g., blood pressure, lipids, Body Mass Index, waist circumference, weight), diabetes self-management behaviors, diabetes knowledge, and psychosocial outcomes (quality of life). Furthermore, the review also investigated the facilitators and obstacles reported by these studies. International guidelines (74, 75) recommend the use of DSME to enhance the health of individuals with diabetes. The ultimate goal is to regulate glycosylated haemoglobin (HbA1c), an outcome noted across all the six studies that used clinical outcome measures.

Studies with positive clinical impact of HbA1c reduction both clinically and statistically (34–38, 49) used various facilitators. Bett’s (49) study achieved HbA1c reduction through a structured education program, follow-up calls, and contextual tailoring of the content. It incorporated Kenyan culture, interactive activities, and the Health Belief Model. Brady et al.’s (37) EXTEND program, based on the UK’s DESMOND, was culturally tailored and co-produced with service users. It helped participants overcome barriers and engage in behavioral and dietary changes. The program was underpinned by several theories and aimed to help participants explore personal risk factors and generate achievable goals. Diriba et al. (34–36) created an intervention based on social cognitive theory and a systematic review they conducted earlier (76). The intervention, designed for participant-caregiver pairs, considered local context, Ethiopian diet, and family concepts. It promoted family involvement in food preparation, physical activities, medication adherence, and blood sugar monitoring. This approach may have led to a significant reduction in HbA1c levels, contradicting their earlier review that showed inconclusive effects of DSME on HbA1c. The results suggest that culturally adapted DSME can be effective for people with diabetes in SSA. Essien et al. (38) conducted a randomised control trial in Nigeria, comparing an intensive, structured DSME program to conventional education. The intensive group showed a statistically and clinically significant reduction in HbA1c levels after six months, with a mean of 8.4%, compared to the conventional group’s mean of 10.2%. Despite the majority of the patients in the conventional group receiving six non-interactive sessions of 30–45 min each, it’s noteworthy that the structured, theme-based, guideline-focused, and resource-intensive interactive sessions can significantly improve HbA1c outcomes.

Although contextual adaptations were key strengths in all 6 studies that measured HbA1c, two studies (8, 46) failed to demonstrate statistical significance due to poor attendance, high attrition, information spillover, and medication shortages. In the study by Hailu et al., only 18% of the intervention group completed all six DSME sessions. Lamptey et al.’s study found a high attrition rate as well. The DSME was run as a one-day, 5-h session. The contact hours needed for a DSME to be effective are still a topic of debate. DSME interventions with 10 h or more contact time have been associated with significant HbA1c reductions (77).

DSME is a fundamental aspect of diabetes management (74). However, in SSA, DSME is often unavailable or of poor quality (32, 76), characterised by *ad hoc*, unstructured information provision (38, 51) or a focus on biomedical models that overlook psychosocial aspects of the illness (30, 78). Systematic reviews and meta-analyses from high-income countries have shown that DSME interventions improve glycaemic control, diabetes knowledge, self-efficacy, quality of life, and mortality rates. However, these results should be cautiously applied to SSA due to cultural, linguistic, religious diversity, and misconceptions (30, 32, 38, 76, 79). Interventions that adhere to guidelines from high-income countries without considering these differences are a barrier to DSME effectiveness. A systematic review and meta-analysis conducted by Diriba et al. (32) suggests the necessity for culturally adapted DSME interventions for Africans living with diabetes. This shift in focus towards cultural adaptation has resulted in positive outcomes in HbA1c levels, as evinced by the DSME interventions included in this review. This shift in improvement is also reflected in the recent systematic review and meta-analysis done by Chowdhury et al. (79) that shows that DSME interventions are effective in LMICs regarding cardiometabolic parameters, diabetes self-management behaviors, and psychosocial wellbeing. This further emphasises the importance of customising DSME interventions to align with the specific characteristics and circumstances of the target audience in SSA. Alaofe et al. (30) also emphasise the importance of incorporating motivation-based health approaches into these culturally tailored DSME interventions.

Another key enabler of a DSME identified in this review is the use of service users or PPI groups in research, as noted across a few studies (34–37, 40, 46) to inform the contextual tailoring of the DSME intervention. PPI group involvement across these studies mainly involved the design of educational resources or checking the contents of the educational programme. Lubega et al. (40) report that the service users were involved in the allocation of study participants to the community clubs according to proximity to their homes. Local foods and cooking practices were included in the EXTEND study (37) as directed by the local PPI group and other topics of importance, such as erectile dysfunction and natural remedies for diabetes. Information on the details and extent of service user involvement in these studies is limited. Although PPI in research is recognised as a valuable tool for improving the quality and relevance of research, it is still in the very early stages of SSA, with many researchers not fully understanding the concept (80). A systematic scoping review by Ankomah et al. (81) found that PPI in SSA was characterised more by tokenism than participation, with implementation activities concentrated on the service design. This observation underscores the gap in service user involvement in research within SSA and emphasises the necessity for additional research. Further investigation is required to explore the impact of culture on service user involvement, as well as to identify barriers and facilitators for successfully integrating PPI in health research.

It is interesting to compare facilitators and barriers to the success and/or failure of the DSME programmes conducted in SSA, reported within this scoping review, to the components suggested by (63). In the UK, a DSME should suit the needs of the person and support the person in developing attitudes, beliefs, knowledge, and skills to self-manage diabetes. This includes a quality-assured structured curriculum that is theory-driven, evidence-based, and resource-effective with supporting materials and is written down and delivered by trained educators. Although findings from this review are similar, notable differences include factors such as program design (content and pedagogy), operational issues impacting workshop attendance, and ‘structural’ (socio-economic) issues impacting users’ abilities to make positive changes to their lives. This includes having to account for barriers such as the weather, accessibility, cost and time of participating in the programme, and cultural beliefs.

The review has emphasised the importance of tailoring DSME interventions to match the characteristics and circumstances of the intended audience. Nurse-led, systematically structured, interactive, group-based, and evidence-based DSMEs for SSA are required. Informed by shared guidelines and theories of learning and behavior change, these contextually tailored DSME have shown positive changes in glycaemic control and other cardiovascular outcomes along with psychological, learning, and behavior outcomes in patients with type 2 diabetes in SSA. The review was not able to report on the impact on staff delivering the programmes and services and highlights a gap in the literature.

## Limitations

This review has several limitations. The first is that the researchers did not submit *a priori* protocol for the review as recommended by JBI (25). Second, a scoping review focus is to provide breadth rather than depth of information in a particular topic, and therefore a meta-analysis of the impact of the nurse-led DSME on primary outcome measures such as HbA1c was not conducted. Third, we limited the search to studies conducted in LMICs in SSA and not globally. This may have impacted the reporting of important themes that addressed the review question and objectives. Third, only ten nurse-led DSME interventions were found in the SSA region. The small number of nurse-led DSME interventions in the SSA region limits the ability to draw robust, generalisable conclusions and may lead to biased or incomplete results due to insufficient data and lack of diversity. Fourth, the majority of the studies reported outcomes from less than a year of follow-up, making it difficult to demonstrate long-term effectiveness. These short-term studies give limited insight into chronic management, and these initial improvements may not hold up in the long run, leading to potentially misleading conclusions about the effectiveness of the interventions and their sustainability. The studies varied in outcome measures, and some were feasibility studies without power calculations. Variability in outcome measures and lack of statistical power in feasibility studies reduce reliability, generalisability, and validity, potentially leading to biased and inconclusive results. Some studies, conducted in urban settings, may not be transferable to rural and remote areas. Urban studies may not be applicable to rural areas due to differences in health care infrastructure, accessibility, cultural practices, resource availability, and generalisability, potentially leading to less effective DSME. Additionally, changes in patients’ medication regimes could have influenced the reduction in HbA1c, questioning the sole effectiveness of the DSME programme. Changes in medication regimes can act as confounding factors, making it difficult to isolate the impact of the DSME program on HbA1c reduction. The majority of the studies reported high baseline HbA1c for patients. This indicates poor glycaemic control, and improvements might be more easily achieved through medication changes rather than the DSME program alone, which limits the assessment of DSME impact. Also, the included studies had different research designs, such as randomised controlled trials, quasi-experimental studies, mixed methods and qualitative interviews. This could have implications for the interpretation of the findings synthesised from the studies. Different research designs lead to variations in data collection and analysis, affecting the reliability and synthesis of findings, and limiting the generalisability of the DSME programme’s effectiveness. Furthermore, the search was limited to four databases, potentially missing relevant studies. Variations in the definition of nurse-led DSME across different studies and countries could also affect the capture of all relevant studies. Unpublished or non-English studies may have been missed. Despite these limitations, we believe this review provides useful information on nurse-led DSME interventions in SSA and how this can be used to inform the development of nurse-led initiatives in the future. Indeed, the researchers have utilised this review to inform the development and delivery of a DSME in Uganda. To overcome these limitations, future research should increase sample size and diversity by conducting more nurse-led DSME interventions across various regions within SSA, including both urban and rural settings. Additionally, designing studies with longer follow-up periods, enhancing study design and rigour by using robust designs such as multicentre randomised controlled trials, standardising outcome measures, and addressing confounding factors will provide more robust and reliable evidence.

### Implications for policy, practice, and future research direction

1. Nurse-led DSME in SSA has demonstrated effectiveness in improving both clinical and nonclinical outcomes for patients. Given resource constraints and the rising prevalence of diabetes, nurse-led structured education plays a crucial role in enhancing patient outcomes and reducing healthcare costs. Stakeholder involvement in the strategic planning of diabetes health systems is required to direct future service delivery.
2. Context-Specific Programmes should be culturally, contextually, and linguistically sensitive to better serve patients in SSA. Adapting interventions to local culture and addressing financial constraints is essential. Future DSME programme development should be coproduced and based on needs analysis of patients and clinicians. The impact on staff in the development of the programmes requires investigation.
3. Structured Educational Package should accompany the theoretical delivery and cover seven key healthcare behaviors related to diabetes management: healthy diet, physical activity, medication adherence, blood glucose monitoring, coping strategies, problem-solving, and foot care. Trained professionals should deliver this structured program and regularly evaluate the impact. Ensuring the educational package remains contemporaneous, reflects best practice, and continues to have a positive impact on the health and well-being of the participants.
4. Theory-Based Education incorporating learning and behavior change theories applicable to the local context is needed. Training educators and measuring the impact through evaluation research of theory-based education are essential.
5. Patient and Public Involvement (PPI) in DSME development has benefits, including better understanding of local needs, improved communication, and enhanced accessibility. Further research should explore the role of service users in DSME design and delivery in the SSA context.

To address the gaps in the research highlighted above, the findings from this scoping review will inform and direct the development, delivery, and evaluation of a nurse-led DSME in Uganda for adults living with T2DM.

## Conclusion

This scoping review sought to answer the following question: What do we already know about the scope of existing practice, nature, and impact of nurse-led diabetic foot prevention services and structured educational programmes on patients with Type 2 Diabetes Mellitus in Sub-Saharan Africa? It is clear that there is a dearth of nurse-led interventions focussed on diabetic foot prevention in SSA; however, there is a small but growing evidence base for nurse-led diabetes self-management education programmes. A consistent message from the analysis of the included papers is that nurse-led, systematic/structured, interactive, group-based, and evidence-based DSME informed by shared guidelines and theories of learning and behavior change adapted to the local needs of patients can have positive changes in glycaemic control. Additionally, there can be improvements in cardiovascular, psychological, learning, and behavioral outcomes. LMICs in SSA need to pay attention to frugal and sustainable initiatives such as nurse-led DSME, which are now needed to address the current epidemic and global health problem of T2DM. Future research exploring the long-term impact of co-produced nurse-led, structured, culturally tailored, and theory-driven DSMEs is needed for future sustainability and effectiveness of these initiatives.

## Data Availability

The original contributions presented in the study are included in the article/supplementary material, further inquiries can be directed to the corresponding author.
